# Supplementary material for: Learning curve and surgical outcome of robotic assisted colorectal surgery with ERAS program
Source: Sci Rep. 2022 Nov 29;12:20566. doi: 10.1038/s41598-022-24665-w (PMC9709162; doi:10.1038/s41598-022-24665-w)
Supplement: Supplementary file 2 — Supplementary Information 2. [file 41598_2022_24665_MOESM2_ESM.zip › RAL Raw data-Ñ[▒K/096/HS_ñw╝╨░O▒Kñσ.pdf]

正式摘要(電子簽章：000000221047232)

W95 / W95 ☐ 英文標題(注意:無英文姓名-請補病人基本檔!) ☐【讀取】憑證

臺中榮民總醫院

出院病歷摘要

轉入號 : 轉出號 :

[redacted]

(8)流水編號	
(9)入院日期	110/07/07 (2021) 直腸外科 W95 - 10
(10)轉科日期	
(11)出院日期	110/07/13 (2021) 住院天數計 6 日

診斷

- 入院 :1. T-colon and rectal adenocarcinoma, at 50cm and 10cm FAV, s/p colonoscopic biopsy on 2021/06/17 at Shin-Der hospital
  2. DM
  3. Renal stone, s/p ESWL
  4. Left proximal radial-ulnar fracture, s/p operation 10+years ago; left distal forearm fracture, s/p operation in 2019
- 出院 :1. T-colon and rectal adenocarcinoma, at 50cm and 10cm FAV, s/p colonoscopic biopsy on 2021/06/17 at Shin-Der hospital, s/p Robotic LAR on 2021/07/08, pT3(2)N1a(1/33)
  2. DM
  3. Renal stone, s/p ESWL
  4. Left proximal radial-ulnar fracture, s/p operation 10+years ago; left distal forearm fracture, s/p operation in 2019

主訴

- loose stool for several months

病史

- This 71-year-old woman has past history of:
  1. T-colon and rectal adenocarcinoma, at 50cm and 10cm FAV, s/p colonoscopic biopsy on 2021/06/17 at Shin-Der hospital
  2. DM
  3. Renal stone, s/p ESWL
  4. Left proximal radial-ulnar fracture, s/p operation 10+years ago; left distal forearm fracture, s/p operation in 2019

  She presented with loose stool for several months. She also had body weight loss of 10kg within 2 years, and decreased appetite. She visited Shin-Der hospital during regular follow up for DM, where colonoscopic biopsy on 2021/06/17 showed adenocarcinoma at 50cm and 10cm FAV. Bloody stool was noted after colonoscopy.
  She then came to our hospital. CEA was 5.33 ng/ml, while CA-199 was 9.11 Unit/ml. Abdominal CT showed a 8.5cm heterogeneous enhancing mass with calcifications over upper rectum with suspect invasion to UB, also, there was another 8 cm heterogeneous enhancing mass over T colon with pericolic fat stranding. There was nodules over S2, S3, S8 of liver; liver cysts are first considered, liver metastasis cannot be ruled out in some nodules. PET on 2021/07/02 reported metastatic lymphadenopathy involving the left presacral region (few) (Grade 4). After discussion, the patient decided to received robotic surgery.
  Under the impression of T-colon adenocarcinoma and rectal adenocarcinoma, the patient was admitted for Robotic LAR scheduled on 2021/07/08.

體檢發現

-
  - General appearance :
    - easy looking , conscious clear , without cardiopulmonary distress
    - Vital signs自動帶入date 體溫(BT) 脈搏(P) 呼吸(R) 血壓(BP) 身高(BH) 體重(BW) BMI
      20210707 36.6C 85/min 18/min 124/64mmHg 169.5cm 56.2Kg 19.6
    - Vital signs: B.P. 124/64 mmHg , P.R. 85 /min , R.R. 18 /min , B.T. 36.6 ℃
  - HEENT :
    - Eyes: no anemic conjunctiva, no icteric sclera, normal EOM
    - ENT: no otorrhea, no rhinorrhea, no erythematous mucosa, no injected tonsil, no deformity
  - Neck: supple, freely movable, trachea midline,no deformity, no engorged jugular vein ,

- no palpable thyroid or lymph node enlargement
- Chest:
  - Inspection: Grossly normal, symmetric and fully expansion, no accessory muscle used, no operation scar, no spider angiomata, no gynecomastia
  - Palpation: no palpable mass or nodule, no subcutaneous emphysema, no tenderness
  - Ausculation: clear breathing sound
- Heart:
  - Palpation: no thrill, no heave
  - Percussion: normal heart size
  - Ausculation: regular heart beat, normal S1, S2, no gallop, no murmur
- Extremities: No deformity, full ranged of motion without limitation, no clubbing finger or nail cyanosis, no pitting edema, no ulcer or pigmentation, no obvious skin lesion, no varicose vein, no bone, joint or muscle tenderness

CRS CONDITION

- Karnofsky performance status: (100) Normal, no complaints, no evidence of disease
- PE of Abdomen:
- __1. Inspection: soft and flat
- __2. Ausculation: normoactive bowel sounds
- __3. Palapation: no tenderness or rebunding pain
- __4. Percussion: dullness
- CEA: 5.33 ng/ml
- Imaging Studies:
- Colonoscope report: adenocarcinoma at 50cm and 10cm FAV
- Sono , CT of abdomen and chest Films:- Abdominal CT showed a 8.5cm heterogeneous enhancing mass with calcifications over upper rectum with suspect invasion to UB, also, there was another 8 cm heterogeneous enhancing mass over T colon with pericolic fat stranding. There was nodules over S2, S3, S8 of liver; liver cysts are first considered, liver metastasis cannot be ruled out in some nodules.
  - PET on 2021/07/02 reported metastatic lymphadenopathy involving the left presacral region (few) (Grade 4).
- Figures:
  1. colon & rectum

手術日期及方法

- 手術日期及方法20210708 Robot Assisted Low Anterior Resection

住院治療經過

- After operation with Robotic LAR on 2021/07/08, the patient was under post-operative care and pain control under PCA use. Self-urination was smoothly after removal of Foley's catheter on 7/11. The patient tolerated clear liquid diet and then Bland No.II diet since 7/10, without N/V, abdominal pain or discomfort. The wound presented with erythematous change with few discharge, r/o wound infection, s/p Unasyn since 7/12. For dirty drainage, r/o chyle leakage, we've tried low fat diet on 7/12, without change of drainage color. Under relative stable condition, the patient was discharge with OPD follow up.

合併症

一般檢查

- 累積報告 :

DATE	TIME	ALB	TP	BIL.T	BIL.D	ALKP	AST	ALT	LDH	AMY	LIPASE	NH3	Ferritin	UA
1100707	1628	3.5		0.21	0.08		11	10						7.5
1100708	2133	2.7		0.45	0.15	63	11	9						

DATE	TIME	WBC	RBC	HGB	HCT	MCV	PLT	BLST	NEUT	BAND	SEG	LYM	MON	EOS	BAS
1100707	1627	6600	3.18	8.6	26.4	83.0	384		70.6			18.8	7.1	2.9	0.6
1100708	2133	8290	3.69	9.8	30.9	83.7	335		90.7			5.4	3.3	0.1	0.5

DATE	TIME	NA	K	CL	BUN	CREAT	eGFR	CA	Mg	P	CRP	ESR	PCT	D-Dimer	LACTATE
1100707	1628	141	4.4		23	1.09		10.9							
1100708	2133	136	4.2	108	13	0.88	67.33	9.5							

DATE	TIME	GLUCOSE	HbA1C	CHOL	TG	HDL	LDL	KETONE	CK	CKMB	TRO-T	PT-P	APTT-P	NT-pro BNP
1100708	2133	214												
1100713	1003				37									

特殊檢查

病理報告

- 活體組織切片病理報告　手術日期：**110/07/08**　病理號：**S1020986**

診斷醫師:楊啟順 報告日期:110/07/16
Pathologic diagnosis:
1. Ascending colon to rectum, robotic low anterior resection --- Adenocarcinoma, moderately differentiated, of rectum. Tumor invaded to perirectal soft tissue; Adenocarcinoma, moderately differentiated, of transverse colon. Tumor invaded to mesocolic soft tissue and metastasized to mesocolic/perirectal lymph nodes (1/22). The bilateral cut ends are free of tumor.
2. IMA root, dissection --- Unremarkable (0/11).
3. Colon, cut end, distal, resection --- Unremarkable.
Ancillary study for diagnosis:
IHC stains for PMS2 and MSH6 are done on section M and N. Both above markers are preserved.
Prognostic and predictive factor:
1. Tumor size: 5.5 cm at rectum; 5.5 cm at T-colon.
2. Depth of invasion: Perirectal soft tissue at rectum; Pericolorectal tissue at T-colon.
3. Histologic grade: G2: Moderately differentiated.
4. Angiolymphatic invasion: Not identified.
5. Perineural invasion: Not identified.
6. Tumor deposits (discontinuous extramural extension): Not identified.
7. Macroscopic tumor perforation: Not identified.
8. Surgical Margins: a) Proximal Margin: Uninvolved by invasive carcinoma, distance of tumor from margin: 16 cm. b) Distal Margin: Uninvolved by invasive carcinoma, distance of tumor from margin: 1.5 cm. c) Radial or Mesenteric Margin: Uninvolved by invasive carcinoma, distance of tumor from margin: more than 1 cm.
9. Lymph node status: Involved (1/33).
10. Extranodal involvement: Not identified.
11. Tumor regression grading S/P CCRT: No known presurgical therapy.
12. Type of polyp in which invasive carcinoma arose: Not identified.
13. Additional pathologic findings: Not identified.
14. Pathological TNM Stage: pT3(2)N1a(According to the eighth edition,
American Joint Committee on Cancer Staging Guidelines for Tumors). 1
15. TNM descriptors: m (Multiple primary tumors).
Gross description:
The specimen consists of 1) a segment of rectum and colon with mesocolic/perirectal soft tissue, 70 cm in length and 9 cm in proximal cut end circumference, 7 cm in distal circumference, and a piece of omentum, 35x15x0.5 cm. A gray tan firm annular elevated and centrally ulcerative tumor, 5.5x5x1 cm, is noted at rectum, 1.5 cm to the distal cut end, and another gray tan polypoid tumor, 5.5x5.5x4 cm, is noted at T-colon, 16 cm to the proximal cut end. On cross section, the tumors invade to the mesocolic/perirectal soft tissue with more than 10 mm to the radial margin. Serosal retraction at T-colon tumor is also noted. The both cut ends are free of tumor grossly. 2) IMA root, a piece of tan yellow soft tissue, 5.5x4x1 cm. 3) distal cut end, a piece of tan mucosa-covered soft tissue, 2x1.5x0.5 cm. Representative part for section: A-B) specimen 2 C) specimen 3 all for section D) distal cut end E) proximal cut end F) omentum G+H) rectal tumor with CFM J-M) rectal tumor N-P) T-colon tumor with serosa Q-T) T-colon tumor U) CFM V-Y) lymph nodes.
Note:
1. The interpretation of MMR panel and recommended studies are as below: (1) No loss of nuclear expression of MMR proteins: low probability of microsatellite instability-high (MSI-H) . (2) Loss of nuclear expression of MLH1 and PMS2: testing for methylation of the MLH1 promoter and/or mutation of BRAF is indicated (the presence of a BRAF V600E mutation and/or MLH1 methylation suggests that the tumor is sporadic and germline evaluation is probably not indicated;
absence of both MLH1 methylation and of BRAF V600E mutation suggests the possibility of Lynch syndrome, and sequencing and/or large

- deletion/duplication testing of germline MLH1 may be indicated). (3) Loss of nuclear expression of MSH2 and MSH6: high probability of Lynch syndrome (sequencing and/or large deletion/duplication testing of germline MSH2 may be indicated, and, if negative, sequencing and/or large deletion/duplication testing of germline MSH6 may be indicated). (4) Loss of nuclear expression of MSH6 only: high probability of Lynch syndrome (sequencing and/or large deletion/duplication testing of germline MSH6 may be indicated). (5) Loss of nuclear expression of PMS2 only: high probability of Lynch syndrome (sequencing and/or large deletion/duplication testing of germline PMS2 may be indicated). (6) Please note: There are exceptions to the above IHC interpretations. These results should not be considered in isolation, and clinical correlation with genetic counseling is recommended to assess the need for germline testing.
  2. This case has been peer reviewed by two doctors.
  #T-59300_2 #M-81403_2 2 1105 S0000F
  #T-C4560_2 #M-81406_2 2 1105 000000

其它

出院時情況

- 3.改門診治療

出院指示

藥名	劑量	劑量單位	用法	頻次	數量	數量單位	現況	天數	單位
Neomycin oint 28gm		TUB	TOP	BID	0	TUB	TH	1	瓶
BethanechoL tab 25mg-JS	1	TAB	PO	TIDAC	21	TAB	TH	7	天
Ultracet tab	1	TAB	PO	Q6H	28	TAB	TH	7	天
(□)Takepron OD tab 30mg	1	TAB	PO	QDAC	7	TAB	TH	7	天
Unasyn TAB 375mg	1	TAB	PO	Q12H	14	TAB	TH	7	天

出院計劃

- 門診日期
  - date on 2021/07/20

主治醫師 2062F 蔣鋒帆 / 住院醫師 2345B 陳映晴

正式摘要**(電子簽章：000000221047232)**

W95 / W95

doctorRank:B
